# Synthesis and Characterization of Proton-Conducting Composites Prepared by Introducing Imidazole or 1,2,4-Triazole into AlPO-5 and SAPO-5 Molecular Sieves

**DOI:** 10.3390/molecules28217312

**Published:** 2023-10-28

**Authors:** Adam Ostrowski, Aldona Jankowska, Agata Tabero, Ewa Janiszewska, Stanisław Kowalak

**Affiliations:** 1 Institute of Molecular Physics, Polish Academy of Sciences, Smoluchowskiego 17, 60-179 Poznań, Poland; 2 Faculty of Chemistry, Adam Mickiewicz University, Uniwersytetu Poznańskiego 8, 61-614 Poznań, Poland; aljan@amu.edu.pl (A.J.); agata.tabero@amu.edu.pl (A.T.); eszym@amu.edu.pl (E.J.)

**Keywords:** proton conductivity, imidazole, 1,2,4-triazole, molecular sieves, AlPO-5, SAPO-5, AFI structure, Brønsted acid centers

## Abstract

The present work concerns proton-conducting composites obtained by replacing the water molecules present in aluminophosphate and silicoaluminophosphate AFI-type molecular sieves (AlPO-5 and SAPO-5) with azole molecules (imidazole or 1,2,4-triazole). Both the introduction of azoles and the generation of Brønsted acid centers by isomorphous substitution in aluminophosphate materials were aimed at improving the proton conductivity of the materials and its stability. In the presented study, AlPO-5 and several SAPO-5 materials differing in silicon content were synthesized. The obtained porous matrices were studied using PXRD, low-temperature nitrogen sorption, TPD-NH_3_, FTIR, and SEM. The proton conductivity of composites was measured using impedance spectroscopy. The results show that the increase in silicon content of the porous matrices is accompanied by an increase in their acidity. However, this does not translate into an increase in the conductivity of the azole composites. Triazole composites show lower conductivity and significantly higher activation energies than imidazole composites; however, most triazole composites show much higher stability. The different conductivity values for imidazole and triazole composites may be due to differences in chemical properties of the azoles.

## 1. Introduction

Crystalline microporous materials are a large group of materials differing in chemical composition, types of bonds, and pore sizes. Their representatives include organic materials, e.g., COF (covalent organic framework), organic–inorganic materials, e.g., MOF (metal–organic framework), and inorganic materials, e.g., zeolites [1]. They have been investigated for a number of potential applications, including gas adsorption (e.g., CO_2_) [2], sensors [3], catalysis [4], energy storage and conversion [5], and many others [6,7,8]. Among inorganic materials, zeolites (natural and synthetic ones) are particularly popular and important since they have been widely used in industrial processes for many years, including catalysis (e.g., Fluid Catalytic Cracking (FCC) and alkylation of aromatic compounds), selective adsorption and separation, water and air purification, and many others [9,10,11]. Zeolites are aluminosilicates composed of AlO_4_ and SiO_4_ tetrahedra linked together at the corners through shared oxygen atoms. The negative charge located in their framework is balanced by extra-framework cations. Importantly, when the charge is compensated by H^+^ cations, bridging hydroxyl groups are formed which can act as Brønsted acid centers, which are very efficient active sites in various catalytic reactions (e.g., FCC) [9,10,11].

The great interest in microporous materials and the resulting intensive development of their synthesis methods have led to the creation of molecular sieves that are analogous to zeolites, known as zeolite-like materials, with various compositions such as AlPO, MeAPO, SAPO, and MeAPSO. The AlPO materials are open-framework aluminophosphates with different crystalline structures composed of alternating AlO_4_ and PO_4_ tetrahedra. The negative charge of the AlO_4_ tetrahedron is compensated by the positive charge of PO_4_. As a result, materials with a neutral framework and low acidity are obtained [12,13,14]. However, their acidity can be improved by isomorphic substitution of the framework atoms. In the case of SAPO materials, which are a combination of AlO_4_, SiO_4_, and PO_4_ tetrahedra, the replacement of some P(V) atoms with Si(IV) atoms results in a negatively charged structure. The presence of a negative charge, similar to zeolites, can lead to the generation of Brønsted acid centers. Another important group of open-framework aluminophosphates is the anionic framework AlPO_4_. For these materials, in addition to quadruple-bridged AlO_4_ and PO_4_ units, other units such as AlO_5_ and AlO_6_ polyhedra, as well as terminal groups such as P–OH and P=O, can be present [15].

The chemical and structural properties of many microporous materials, including their ability to adsorb molecules in their pores, make them suitable hosts for the dispersion of molecules capable of being involved in the transport of H^+^ ions. It has been shown that the introduction of water or azole molecules into microporous materials can lead to obtaining materials exhibiting even higher proton conductivity than the porous materials themselves [16,17,18]. Materials formed in this way can be considered as potential solid electrolytes in PEMFC (Proton-Exchange Membrane Fuel Cells)—very promising devices for generating electrical energy without emitting environmentally harmful substances which are currently used, among other things, as power sources. Crystalline microporous materials have been repeatedly tested for their possible use in solid electrolytes in PEMFCs. So far, much attention has been paid to proton conductors representing a variety of MOF and COF materials containing water molecules or azoles [19,20,21,22], with materials tested in conditions of high relative humidity being by far the most common object of research. In contrast, there have been relatively few literature reports concerning materials based on zeolites [16,17,18] and open-framework aluminophosphates or silicoaluminophosphates. Proton conductivity in the presence of water molecules has been tested in the aluminophosphates with a neutral [23] and anionic framework [24,25,26] as well as in silicoaluminophosphates [27]. In the case of andydrous proton-conducting zeotype–azole materials, imidazole-loaded AlPO-5 [17], an aluminophosphate with imidazole obtained by tribochemical method [28], and imidazolium imidazole-templated aluminophosphate [29] have also been tested. The presented studies have motivated us to search for new materials that could act as proton conductors.

This work concerns proton-conducting composites obtained by introducing azoles into aluminophosphates and silicoaluminophosphates with the AFI structure. As part of our initial research on azole–zeotype systems, it was established that the composite combining nonpolar AlPO-5 and imidazole exhibits lower and less stable conductivity compared to composites based on polar zeolites with different structures [17]. These observations allowed us to assume that the parameters of the azole composite may depend to some extent on the acidity of the porous matrix. For this reason, the aim of the presented work was to determine whether the generation of Brønsted acid centers by introducing Si atoms into aluminophosphate materials contributes to the improvement of proton conductivity of azoles composites and its stability. Therefore, in this study, we investigated proton conductors obtained on the basis of AFI-type porous materials differing in acidity (AlPO-5 and SAPO-5) and azoles (imidazole and 1,2,4-triazole). In the research, SAPO materials with different silicon content and different numbers of acid centers were used. The obtained results may contribute to the expansion of knowledge regarding proton-conducting solid electrolytes combining zeolite-like materials and azoles: imidazole and, unlike in previous works, 1,2,4-triazole.

## 2. Results

### 2.1. Structure

**Figure 1:**
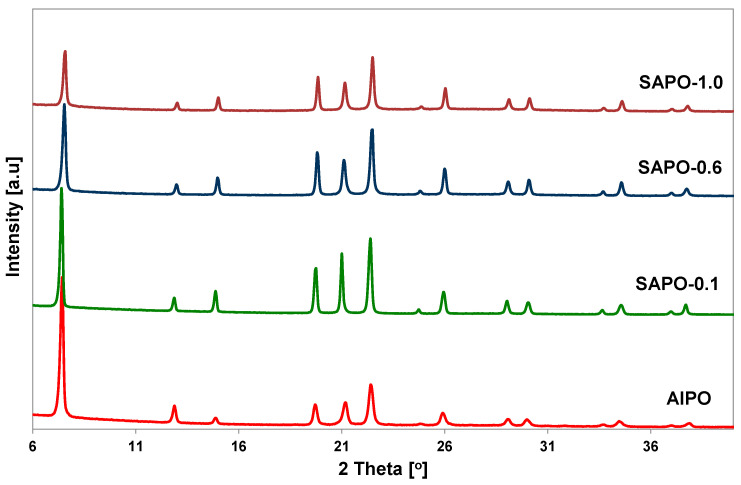
Powder XRD patterns of the obtained AlPO-5 and SAPO-5 matrices with different Si content.

PXRD analysis was performed to confirm the correctness of the structure and evaluate the degree of crystallinity of the obtained porous matrices. The crystallinity of the samples was assessed by examining the intensity of XRD diffraction lines attributed to the (100), (210), (002), and (102) planes of the AFI structure. The relative intensities of the diffraction lines are summarized in Table 1. Powder XRD patterns of all obtained samples showed well-resolved reflections characteristic of AFI structure (Figure 1).

No additional peaks were observed, indicating that the samples were free from impurity phases. The introduction of a small amount of silicon source into the gel caused an increase in the crystallinity of the obtained samples. However, with an increase in silicon content, a decrease in the crystallinity of the SAPO materials was observed, as demonstrated by the calculated degree of crystallinity and X-ray diffraction patterns (Table 1, Figure 1). These results indicate that the introduction of a relatively large amount of silicon disrupts the crystallization process and leads to obtaining a material with reduced crystallinity. Similar observations have been described in the literature [30].

With an increase in silicon content, there was also a shift of reflections towards higher 2theta values, accompanied by an increase in the overall unit cell volume, which is in line with the literature data [31]. The unit cell parameters calculated for the SAPO samples were compared with the data for AlPO and are summarized in Table 1. Only the material with the highest silicon content had a unit cell volume lower than AlPO sample.

### 2.2. Morphology

The addition of silicon atoms to the AFI framework not only generates Brønsted acid sites, as mentioned earlier, but can also influence the morphology of the samples [32,33,34]. Molecular sieves with uniform, molecular-sized pores are characterized by excellent thermal, mechanical, and chemical stability, making them suitable for potential applications in membrane-based separation and catalysis [35,36]. The microstructure of membranes, including continuity, crystal orientation, and thickness, is of great importance in real applications [37]. The production of membranes or sensors requires crystals with specific size, shape, and orientation, and electron microscopy is often used for their characterization [38,39,40].

**Figure 2:**
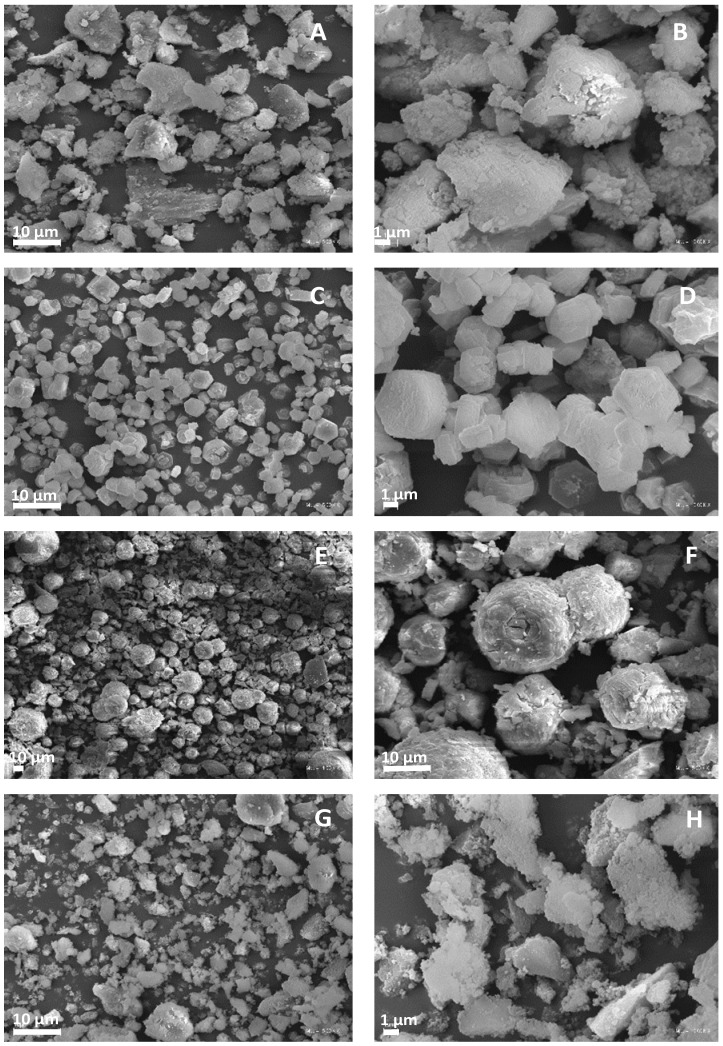
SEM micrographs of different magnitude for obtained matrices: AlPO (**A**,**B**), SAPO-0.1 (**C**,**D**), SAPO-0.6 (**E**,**F**), and SAPO-1.0 (**G**,**H**).

Microscopic images of the obtained matrices are presented in Figure 2. As can be seen, the AlPO and SAPO-1.0 samples exhibited similar morphology. They possessed poorly crystallized, irregularly sized crystallites with blurred edges. These results are consistent with the PXRD results. In the case of the remaining samples, significant differences in morphology were visible, which may result from different chemical compositions and crystallization conditions (Table 2) [32,41,42,43]. In the case of SAPO-0.1 and SAPO-0.6, as the silicon content increased, the morphology changed from hexagonal plates to spherical aggregates. Moreover, the SAPO-0.1 exhibited the most well-developed crystals, as confirmed by the PXRD results.

### 2.3. Textural Properties

**Figure 3:**
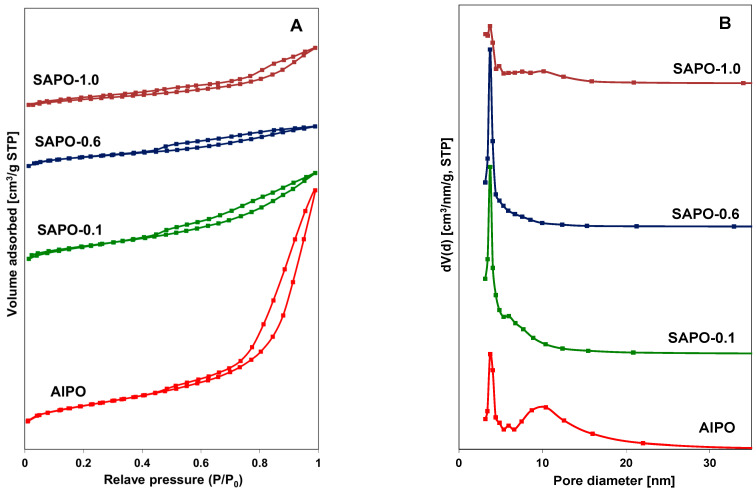
N_2_ adsorption/desorption isotherms (**A**) and pore size distribution (**B**) for indicated materials.

Nitrogen adsorption–desorption isotherms and the pore size distribution of AlPO and SAPO materials are shown in Figure 3A,B. The obtained type IV isotherms exhibited hysteresis loops at a relative pressure (P/P_0_) above 0.4, which may indicate that the obtained materials have a similar character to mesoporous zeolites [44,45]. The presence of a hysteresis loop between the values of P/P_0_ = 0.8 and 1 in the AlPO material indicated the presence of mesopores. This observation may be associated with the contribution of an amorphous phase (Figure 3A). This material also had the largest pore diameters of approximately 10 nm. The average pore diameter for SAPO materials was about 3–4 nm. The pore size distribution curves (Figure 3B) indicate that, in the case of the AlPO material, pores bigger than 6 nm predominated, whereas for the SAPO materials, particularly SAPO-0.1 and SAPO-0.6, pores with a diameter of approximately 3.7 nm were predominant. The textural properties of the obtained matrices are summarized in Table 3.

The obtained results indicate a slightly smaller total surface area (S_BET_) of silicon-containing materials compared to the AlPO material. As the silicon content increased (up to 0.6), an increase in the contribution of micropores was also observed, while the surface area of mesopores decreased. The presence of silicon also significantly affects the pore volume. Materials containing silicon have a nearly threefold smaller total pore volume. Moreover, the micropore volume is inversely proportional to the mesopore volume and increases with increasing silicon content.

### 2.4. Infrared Spectroscopy

The introduction of silicon into the framework of aluminophosphate with AFI structure was also confirmed by FTIR infrared spectroscopy. Table 4 presents the bands occurring in zeolites and zeolite-like materials [46,47].

**Figure 4:**
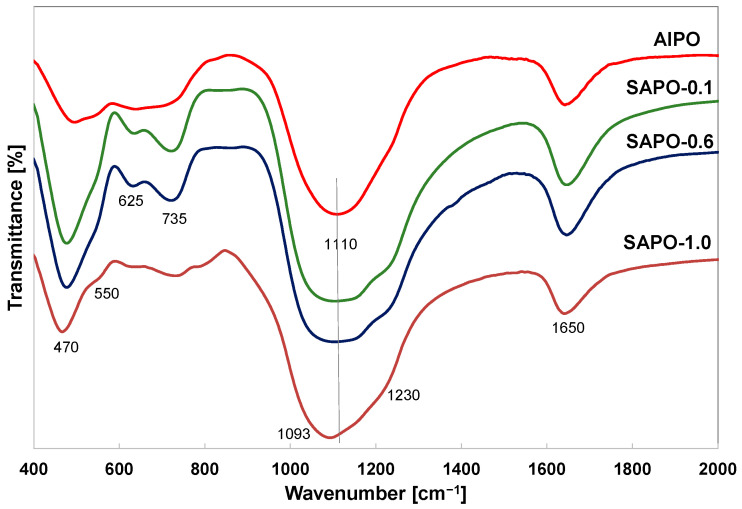
FTIR spectra of indicated samples.

The spectra of obtained matrices showed characteristic bands for the vibrational frequencies of the T–O–T units (T = Si, Al, and P) and vibrations present in the secondary building units D4R and D6R of the AFI structure (Figure 4). The band at ~1650 cm^−1^ is associated with the bending vibration of H–O–H [46,47,48]. Despite the different morphologies of the samples, the spectra exhibited absorption bands located in similar positions. In the case of matrices with lower crystallinity (AlPO, SAPO-1.0), lower intensity of the bands (~625, 735 cm^−1^) sensitive to structural changes in tetrahedra was observed.

The broad band around 1000–1250 cm^−1^ is attributed to the asymmetric stretching vibration of T–O and is characteristic of all zeolites and zeolite-like materials [46,47,48]. The intensity of this band was the higher for the SAPO-0.1 and SAPO-0.6 samples. With an increase in silicon content, this band shifts towards lower frequencies. According to the literature data, this band can shift towards lower wavenumbers with an increase in aluminum content in the unit cell of a zeolite composed of aluminum and silicon tetrahedra [49,50] or to higher frequencies in SAPO materials compared to aluminosilicate materials due to the presence of significant amounts of phosphorus [51]. The shift of this band towards lower frequencies in the spectra of presented SAPO materials may suggest that silicon atoms substitute mainly for phosphorus atoms, and this is due to the different bond lengths in the tetrahedra. The bond length of P–O (1.54 Å) is smaller than the bond length of Si–O (1.61 Å) or Al–O (1.75 Å) in zeolites [51].

### 2.5. The Acidity of Matrices

According to the literature data, in the case of aluminophosphate materials, replacing phosphorus atoms with silicon atoms leads to the generation of Brønsted acid sites with a medium strength similar to that of NaHY zeolite [52,53,54,55,56].

**Figure 5:**
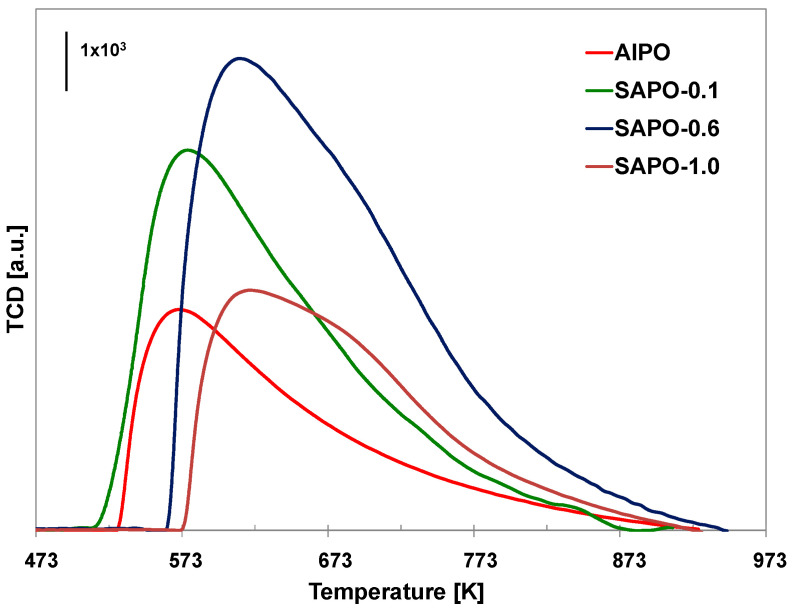
TPD-NH_3_ profiles of the obtained materials.

The results of the TPD-NH_3_ analysis conducted on the obtained materials are presented in Table 5 and Figure 5. Incorporating silicon atoms into the aluminophosphate framework leads to the formation of additional acid sites, causing an increase in the total number of acid centers. Moreover, as silicon content increases, the number of both weak (LT) and strong acid centers (HT) increases. An exception was the sample with the highest silicon content (SAPO-1.0), which may be due to the lower crystallinity of this material. According to the literature data, conducting the synthesis of SAPO materials at low pH leads to the substitution of one phosphorus atom with one silicon atom, generating weak Brønsted acidic centers. At high pH, in contrast, two Si^4+^ atoms replace one Al^3+^ and one P^5+^ atom, generating strong acid centers [57]. Despite conducting the synthesis at a low pH, the SAPO materials obtained in our study mainly possessed strong acid centers. It could have resulted from applying reagents other than those used by the authors of the above-mentioned work. As our research shows, attempts to introduce a relatively large amount of silicon disturb the crystallization process and may result in the incorporation of a lower amount of silicon than is present in the crystallization gel, which may explain the decrease in the number of acidic centers for SAPO-1.0.

The introduction of silicon atoms into the framework of the obtained SAPO materials was also confirmed by their catalytic activity in the esterification reaction of acetic acid with n-butanol. The AlPO material was not active in the esterification reaction, while the SAPO materials showed activity in this reaction (AlPO—0%, SAPO-0.1—45%, SAPO-0.6—19%, SAPO-1—10%). This confirms the incorporation of silicon atoms into the framework, resulting in the formation of Brønsted acidic centers.

### 2.6. Proton Conductivity

**Figure 6:**
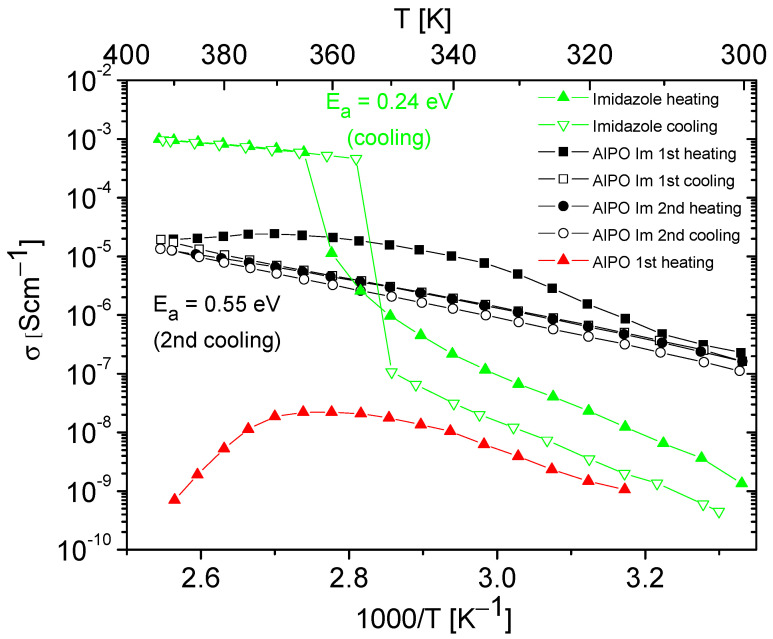
Temperature dependencies of the conductivity of imidazole, AlPO matrix, and its composite containing imidazole obtained during the heating–cooling cycle. The activation energy values were determined from cooling cycle.

**Figure 7:**
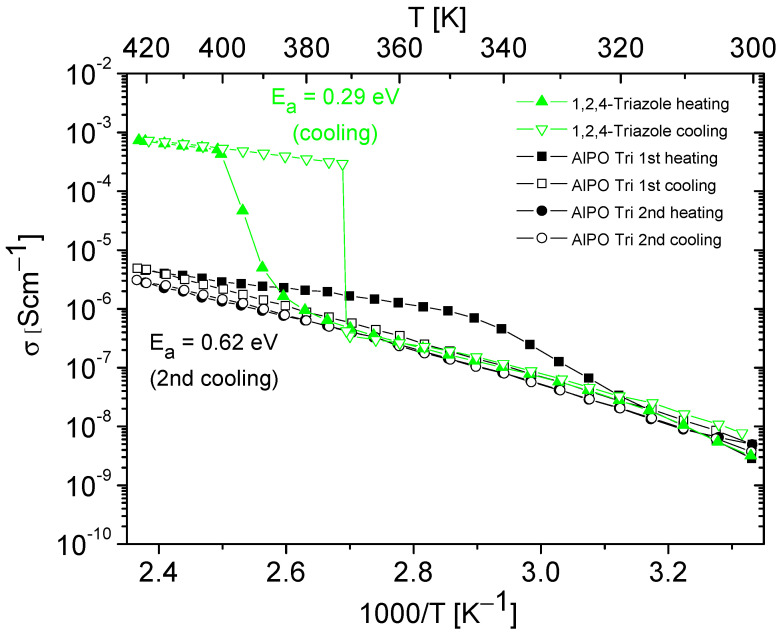
Temperature dependencies of the conductivity of 1,2,4-triazole and AlPO containing 1,2,4-triazole, obtained during the heating–cooling cycle. The activation energy values were determined from cooling cycle.

The conductivity measurements were performed for polycrystalline azoles, the matrices, and the obtained composites. Polycrystalline imidazole and 1,2,4-triazole show low conductivity values at room temperature (about 10^−8^–10^−9^ S/cm^−1^), which are associated with the limited mobility of azole molecules in the crystalline lattice in the solid phase [58]. The conductivity of azoles increases with rising temperature and then experiences rapid growth by several orders of magnitude at the temperature corresponding to their melting point (Figure 6 and Figure 7). At the phase transition (from solid to liquid phase), the mobility and reorientation of azole molecules significantly increase. Additionally, there is the possibility of breaking and forming new hydrogen bonds between neighboring azole molecules, resulting in effective proton transport and, consequently, a significant increase in proton conductivity in this phase [59].

AlPO-5 and SAPO-5 materials, like other porous materials, naturally contain a certain number of water molecules trapped within their pores. Although hydrated matrices may exhibit relatively high conductivity values at room temperature, with increasing temperature, there is a rapid loss of weakly bound water molecules from the matrix, leading to a significant conductivity decrease [16,17,58,60,61,62,63,64]. We observed similar phenomena for AlPO and SAPO materials. An example of the conductivity temperature dependence for an AlPO matrix, naturally containing water molecules, is shown in Figure 6. Our measurements of proton conductivity for AlPO and all SAPO matrices not filled with azoles revealed that after the first heating cycle, the conductivity at high temperatures dropped to values below ~10^−9^ S/cm^−1^ and was unmeasurable in the second cycle of heating–cooling.

Completely different conductivity trends were observed for azole composites. In the studied composites, water molecules were replaced by azole molecules, which have much higher boiling points (~533 K). As a result, these composites maintained high conductivity at temperatures exceeding 373 K, making them potentially suitable for use in conditions favorable for the effective operation of the fuel cells. Below, we discuss the conductivity of composites combining porous matrices (AlPO or SAPO with different silicon contents) and azoles (imidazole or 1,2,4-triazole).

**Figure 8:**
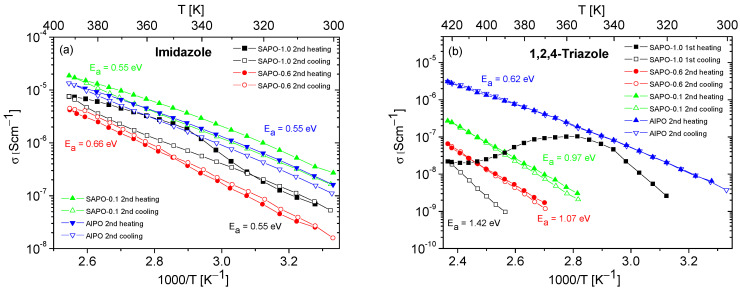
Temperature dependencies of the conductivity of composites containing imidazole (**a**) or 1,2,4-triazole (**b**), obtained during the heating–cooling. The activation energy values were determined from the second cooling cycle (from first cooling cycle for SAPO-1.0).

The temperature-dependent conductivity measurements for all composites were performed for two heating–cooling cycles in temperature ranges 300–393 K and 300–423 K for imidazole and triazole, respectively. In Figure 6, Figure 7 and Figure 8, in addition to conductivity profiles, the activation energy values determined from the second cooling cycle are also included (unless otherwise mentioned). Composites with various azole contents were investigated, but only the results for materials with an azole loading of 0.2 wt.% are presented as this loading proved to be the most beneficial in terms of filling the matrix pores and the obtained conductivity values. At higher azole concentrations, some of the imidazole or triazole molecules were located outside the pore system, on the outer surface of the matrix, as indicated by the phase transition effect recorded in the conductivity measurements. This effect is similar to that observed for polycrystalline azoles. On the other hand, in the case of lower azole contents, the composites showed correspondingly lower conductivity values. Such behavior was also observed in our previous studies [17,58,63,64].

Figure 6 and Figure 7 present the results of the conductivity measurements for AlPO matrix and its composites containing imidazole or 1,2,4-triazole as a function of temperature. In both types of composites, a notable decrease in conductivity was evident during the first heating–cooling cycle, which can be attributed to the loss of a certain number of azole molecules. In contrast to the composite with imidazole, the one with triazole exhibited stable conductivity values in the second heating–cooling cycle; however, its conductivity values were approximately five times lower.

The results of conductivity measurements for composites based on SAPO materials with different Si content are presented in Figure 8. Similarly to the composites obtained with AlPO (Figure 6 and Figure 7), a significant decrease in the conductivity values was also observed for SAPO composites during the first heating–cooling cycle. For this reason, and to enhance clarity, only the results for the second heating–cooling cycle are shown in Figure 8. The exceptions are the data for the SAPO-1.0 with triazole, where only the first heating–cooling cycle is shown in Figure 8b. In the case of this composite, similar to matrices containing only water molecules (Figure 6), a significant decrease in the conductivity values was observed for the first measurement cycle, and the conductivity in the second cycle was unmeasurable.

For composites containing 1,2,4-triazole, lower conductivity values and higher activation energies were recorded for all samples compared to for composites containing imidazole. Among the triazole-based composites, the one based on the AlPO matrix exhibited the highest and most stable conductivity. With an increase in silicon content in the matrix, a significant decrease in the conductivity of the composites and a substantial increase in activation energy values were observed.

Although the conductivity values obtained for composites with triazole were much lower compared to those obtained for composites containing imidazole, the course of the second heating–cooling cycle showed their higher stability (except for the SAPO-1.0 composite) compared to composites with imidazole. The only relatively stable imidazole composite was that based on the SAPO-0.6 matrix. The reasons for these observations can be found in the different physical and chemical properties of the applied matrices.

## 3. Discussion

In the earlier work, we used both polar (aluminosilicate) and nonpolar (aluminophosphate) matrices to obtain proton-conducting composites [17]. Both low- and high-silicon zeolites proved to be effective matrices for preparing proton-conducting composites. The lowest conductivity in this study was recorded for the nonpolar material AlPO. Moreover, we have shown that the presence of Brønsted acid centers, particularly those of weak and moderate strength, can be advantageous for proton conductivity [63,64]. Bearing this in mind, in the case of SAPO materials, it was assumed that the incorporation of silicon into the AlPO material would generate Brønsted acid centers that would act as additional proton carriers, thus increasing conductivity. Therefore, both an increase in conductivity (due to the increased number of protons originating from the acid centers and the ability of additional acid centers to act as proton carriers) and the formation of stable bonds between azole molecules and the matrix surface were expected. The results presented in Table 5 show that the acidity of SAPO materials was higher than that of AlPO. Moreover, the formation of Brønsted acidic centers in SAPO was confirmed by their catalytic activity in the esterification reaction of acetic acid with n-butanol (Section 2.5). However, the increase in acidity did not translate into an increase in the conductivity of the azole composites. These observations may be related to the number of centers in the porous matrices.

To obtain composites with stable and high conductivity, the azole–matrix bonds should both allow free reorientation of azole molecules and be durable enough to prevent desorption of azoles from the matrix pores. Therefore, the crucial factor ensuring stability and high conductivity should be the optimal bonding strength and number of acid centers per unit surface [58,63,64]. Unfortunately, the analysis of the data presented in Table 5 reveals that the number of acid centers per SBET surface area of the investigated composites was relatively low (compared to previously studied materials), both in the AlPO material (as expected) and its SAPO derivatives. For imidazole-based composites, only in the case of the composite based on the SAPO-0.6 matrix were relatively stable conductivity properties obtained, which can be explained by the highest number of acid centers among all examined matrices (Table 5).

Another reason for the lack of desired stability of the composites may be the structural parameters of the used porous matrices. Porosity analysis (Figure 3 and Table 3) revealed that the average diameters of both micropores and mesopores in the examined materials were much larger than the size of azole molecules (~ 0.4 nm) [65]. Such matrix parameters allow for the uptake of a high number of azole molecules into the pores. On the other hand, they do not provide a durable bonding of all adsorbed azole molecules on the available pore surface. Therefore, as our previous research [58] has shown, the crucial factor for optimal filling of matrices with azole molecules and obtaining stable and highly conductive composites seems to be not so much the large available pore volume, but rather the size and chemical properties of the surface. The investigated materials, AlPO and SAPO, possess a relatively large pore volume and surface area, but the number of their acid centers is very limited. As a result, at high temperatures, easy desorption of a portion of azole molecules from the surface of most studied composites can occur.

As shown in Figure 8b, in the case of triazole composites, conductivity values decreased (beginning from AlPO) as the Si content increased. As mentioned earlier, this phenomenon cannot be explained only by acidity values, which were relatively low for all studied matrices. The analysis of the texture parameters collected in Table 3 reveals that, as the Si content increased, in addition to a reduction in pore volume, an increase in the ratio of micropore area to mesopore area was also observed. It is worth highlighting that for all matrices the SBET values were comparable.

Considering the above results, it can be assumed that during the preparation of the composites, not all triazole molecules can be located on the surface of the micropores, and a significant number of them are also incorporated outside the pore system. Such composites could lose a substantial amount of triazole in the first heating–cooling cycle, resulting in relatively low conductivity values due to the reduced number of proton carriers. In the case of composites with imidazole, a similar relationship is not observed, and the studies carried out have not provided a clear explanation.

It seems that the differences in conductivity between composites containing imidazole and triazole may arise from the different chemical properties of the azole molecules. The imidazole molecule is a stronger Brønsted base and can, therefore, react with both strong and weak Brønsted acid sites, while triazole is a weaker base and mainly reacts with strong acid centers. Consequently, due to its more basic character, imidazole may be more capable of deprotonating OH groups of the Brønsted acid centers, leading to the formation of protonated azole molecules. A higher number of formed imidazolium cations, in turn, can enhance proton transport efficiency, as indicated by the lower activation energies determined for imidazole composites, and consequently increase the conductivity of imidazole composites. On the other hand, due to their high dynamics, imidazole molecules are less strongly bound to the internal surface of the matrix, resulting in the lack of stable proton-conducting properties in imidazole composites. It is also worth noting that there are differences in the conductivity values of the applied azoles. The conductivity value in the high-temperature phase for polycrystalline imidazole is higher than for 1,2,4-triazole (Figure 6 and Figure 7). It may also affect the resultant conductivity of the composites.

## 4. Materials and Methods

### 4.1. The Preparation of AlPO-5 and SAPO-5

The materials used as matrices to prepare the composites were synthesized according to the procedure described in the literature [31,66] with some modification. In the case of SAPO materials, samples with different silicon content were obtained. Details of the syntheses are given in Table 2.

Aluminum isopropoxide (ACROS, Waltham, MA, USA), phosphoric acid (85%, POCh, Gliwice, Poland), and tetraethyl orthosilicate (TEOS, Sigma-Aldrich, St. Louis, MO, USA) were used as precursors of Al, P, and S, respectively. Triethylamine (TEA, Sigma Aldrich) was used as the structure-directing agent (SDA) to obtain materials with the AFI structure.

In a typical synthesis, aluminum isopropoxide is aged before use. For this purpose, half the amount of distilled water required for synthesis was added to the aluminum source and stirred for 2 h. The resulting suspension was left under static conditions at room temperature for 24 h. Then, in the case of the SAPO synthesis, the required amount of tetraethyl orthosilicate dissolved in ethanol was slowly added, and the obtained suspension was stirred for one hour. In the next stage, orthophosphoric acid diluted with the required amount of water was added and stirred for another hour. In the final synthesis step, the necessary amount of TEA was added and stirred for an additional hour. The pH value of the starting gel was adjusted by adding an additional amount of H3PO4 acid. Crystallization was carried out in stainless steel autoclaves and heated to the required temperature. The obtained product was washed several times with distilled water and dried at 333 K. The product was calcined at 823 K for 8 h to remove the template. The temperature ramp rate up to 823 K was 1 K/min.

### 4.2. The Preparation of Azole Composites

Composites containing azoles were prepared by impregnation of molecular sieves with solutions containing imidazole (Aldrich) or 1,2,4-triazole (Aldrich). Before impregnation, the matrices underwent thermal activation (623 K, 3 h) to remove adsorbed water. Subsequently, solutions of imidazole (in chloroform) or triazole (in methanol) were added to the activated matrices. The mass fraction of azole in each presented composite was 0.2. The mass fraction of azole was determined experimentally, and in each composite was 0.2. Molecular sieve suspensions in azole solutions were mixed in closed vials for 24 h at room temperature. Then, the solvent was evaporated under stirring at 308 K.

### 4.3. Characterization

Measurements of PXRD (Powder X-ray Diffraction) were performed using the Bruker AXS D8 Advance instrument (Bruker, Billerica, MA, USA) equipped with a Johansson monochromator (αCu Kα1 = 0.15406 nm) in the range of 5° < 2θ < 55° with a step size of 0.02°. Based on the diffraction data, the degree of crystallinity of the samples was determined using Equation (1):

% Crystallinity = (∑I/∑Is) × 100(1)

where: I—intensity of the sample line, Is—intensity of the standard sample line.

The standard sample was the sample with the highest crystallinity. In the calculations, the intensity of reflections at 7.5°, 14.9°, 19.8°, 21.1°, 22.5°, and 26.0° 2 theta angles was taken into account [67].

The textural properties of the matrices were determined using the low-temperature N_2_ adsorption/desorption method, using the NOVA 1000e device (Quantachrome, Boynton Beach, FL, USA). Before measurement, the samples underwent evacuation at 573 K for 12 h. Adsorption/desorption was carried out by passing nitrogen through the sample, which was maintained at the temperature of liquid nitrogen. The surface area and pore size distribution were calculated using the Brunauer–Emmett–Teller and Barrett–Joyner–Halenda methods, respectively. The micropore volume and external surface area were determined using the t-plot method. The micropore surface area was calculated as the difference between the total and external surface area. The total pore volume was estimated using the single-point model (at P/P_0_ = 0.98).

The esterification reaction of acetic acid with n-butanol was conducted in sealed glass vials. For this reaction, 0.05 g of catalyst was used, which was previously activated in an oven at 623 K for 1 h to remove the water accumulated in the pores of the catalyst. To the weighed catalyst, 1207 g of glacial acetic acid (Carlo Erba, Emmendingen, Germany) and 2965 g of n-butanol (Eurochem, Zug, Switzerland) were added (the molar ratio of acetic acid to n-butanol was 1:2). The reaction was carried out in a thermostatic bath with continuous stirring at 373 K for 4 h. The activity of the catalyst was estimated by titrating the mixtures obtained after the reaction with 0.1 M sodium hydroxide solution, allowing for the calculation of the amount of unreacted acetic acid. Prior to analysis, the obtained mixtures were centrifuged to separate the catalyst from the reagent mixture. Then, 0.5 cm^3^ of the resulting solution was transferred to a volumetric flask and diluted with 10 cm^3^ of water. Phenolphthalein was used as the indicator for the alkalimetric titration.

The amount and strength of acidic centers were determined using temperature-programmed desorption of ammonia (TPD-NH_3_). Measurements were carried out on a PulseChemiSorb 2705 device (Micromeritics, Norcross, GA, USA) with a flow system. The sample (~500 mg) was purged with a stream of helium (Linde, Munich, Germany), with the temperature gradually increasing (10 K/min) up to 623 K, and then held at this temperature for 30 min. Then, the tested sample was cooled to 393 K and saturated with ammonia for 0.5 h. Physically adsorbed ammonia was removed in a stream of helium (50 cm^3^/min) at 393 K for 1 h. The analysis was performed in the temperature range of 393–1173 K with a heating rate of 10 K/min. Desorbed ammonia was detected using a TCD detector. All TPD-NH_3_ profiles presented in this study were normalized to the sa sample mass (i.e., 1 g).

The morphology of the matrices was estimated using scanning electron microscopy (SEM, Hitachi SU3500, Hitachi, Tokyo, Japan).

Proton conductivity of azoles, obtained matrices, and composites was measured using impedance spectroscopy in a temperature-programmed system (1 K/min) in the range of 300–393 K for composites with imidazole and 300–423 K for composites with triazole. Powdered samples were compressed between electrodes in a Teflon vessel (with a diameter of 8 mm and a sample thickness of 1.5–1.8 mm) under a pressure of 200 MPa. The measurements were carried out in a nitrogen atmosphere using a precise LCR 4284A Hewlett Packard meter (20 Hz–1 MHz). The measurement temperature was controlled and stabilized using a LakeShore 340 temperature controller (Lake Shore Cryotronics, Inc., Westerville, OH, USA).

## 5. Conclusions

To date, the research on proton conductors based on zeolites or zeolite-like materials has mainly focused on materials that exhibit water-mediated proton conduction. These solid electrolytes typically require a high-humidity environment to function effectively in fuel cells. In our current paper, we show that replacing water molecules present in AFI-type molecular sieves with azole molecules increases the conductivity of the materials and its stability. Moreover, our research demonstrates that the type of azole used affects the conductivity value of composites. In the case of composites containing 1,2,4-triazole, all samples have lower conductivity values and higher activation energies compared to composites containing imidazole, but they can operate at even higher temperatures. This is desirable and allows for higher efficiency of the fuel cell.

Among the composites showing relatively stable conductivity, the highest and most stable conductivity was observed in the composite based on the AlPO matrix with 1,2,4-triazole. For composites containing imidazole, the highest conductivity (although not very stable) was recorded for the SAPO-0.1-based sample. On the other hand, the matrix providing the highest stability for the imidazole-containing composite was SAPO-0.6, which was the porous matrix with the highest acidity.

The lower-than-expected conductivity values of the obtained composites may be related to the relatively low number of acid centers per surface (expressed as A/S) in both the AlPO material and its SAPO derivatives. As a result, at high temperatures, azole molecules can be easily desorbed from the surface of most investigated composites. Furthermore, the introduction of Si during synthesis leads to changes in other parameters (structural—decrease in crystallinity or textural—reduction in surface area, pore volume, and a change in the micro-to-mesoporous surface ratio), which can also affect the conductivity.

## Figures and Tables

**Table 1:** Crystallographic data and degree of crystallinity for AlPO-5 and SAPO-5 with different Si content.

Sample	a = b (Å)	c (Å)	Volume (Å^3^)	Crystallinity (%)
AlPO	13.6911	8.3764	1360	54
SAPO-0.1	13.7314	8.4398	1378	100
SAPO-0.6	13.7892	8.4638	1394	58
SAPO-1.0	13.5314	8.4040	1333	43

**Table 2:** Chemical composition of synthesis gels and condition of synthesis.

Sample	Gel Composition					pH		Temperature (K)	Time (h)
	Al_2_O_3_	SiO_2_	P_2_O_5_	TEA	H_2_O	Before	After		
AlPO	1	0	1	1	40	4.3	7.0	423	23
SAPO-0.1	1	0.2	1	1	40	5.1	9.8	448	22
SAPO-0.6	1	1.2	1	1	40	5.0	9.7	448	22
SAPO-1	1	2	1	1	40	5.2	9.8	448	22

**Table 3:** The textural parameters of AlPO and SAPO materials.

Sample	S_BET_^a^ (m^2^/g)	V_t_^b^ (cm^3^/g)	V_micro_^c^ (cm^3^/g)	V_meso_^d^ (cm^3^/g)	S_ext_^e^ (m^2^/g)	S_micro_^f^ (m^2^/g)	S_micro_/S_meso_
AlPO	266	0.58	0.048	0.532	168	98	0.58
SAPO-0.1	251	0.27	0.063	0.207	126	125	0.99
SAPO-0.6	255	0.22	0.117	0.103	76	179	2.36
SAPO-1.0	242	0.23	0.085	0.145	73	169	2.32

^a^ specific surface area, ^b^ total pore volume, ^c^ micropore volume, ^d^ mesopore volume (V_t_ − V_micro_), ^e^ mesopore surface, ^f^ micropore surface.

**Table 4:** Infrared bands assignments to vibrations in zeolites and zeolite-like materials [46,47].

Vibrations	Internal Tetrahedra (cm^−1^) (Structure Insensitive)	External Linkages (cm^−1^) (Structure Sensitive)
ν_as_ (T–O–T)	1250–950	1050–1150
ν_s_ (T–O)	720–650	820–750
Double ring	-	650–500
T–O bend	500–420	-
Pore opening	-	420–300

T = Si, Al, P.

**Table 5:** The number and strength of acid centers estimated from the results of TPD-NH_3_.

Sample	Total Acidity (µmol/g)	LT ^a^	HT ^b^	LT/HT	A/S ^c^ (µmol m^−2^)
AlPO	91.8	50.7	41.1	1.23	0.35
SAPO-0.1	142.8	74.6	68.2	1.09	0.57
SAPO-0.6	224.9	96.5	128.4	0.75	0.88
SAPO-1.0	120.3	49.1	71.2	0.69	0.49

^a^ LT (low temperature)—weak acid sites, max. ˂ 653 K; ^b^ HT (high temperature)—strong acid sites max. > 653 K; ^c^ A/S—number of formed acid sites (A) related to the surface unit (S).

## Data Availability

The data presented in this study are available on request from the corresponding author.
